# No moment wasted: the primary-care visit for adults with diabetes and low socio-economic status

**DOI:** 10.1017/S1463423615000134

**Published:** 2015-05-20

**Authors:** Shari D. Bolen, Paulette Sage, Adam T. Perzynski, Kurt C. Stange

**Affiliations:** 1 Department of Medicine, Center for Health Care Research and Policy, Case Western Reserve University, MetroHealth Medical Center, Cleveland, Ohio, USA; 2 Department of Epidemiology and Biostatistics, Case Western Reserve University, Cleveland, Ohio, USA; 3 Department of Sociology, Case Western Reserve University, Cleveland, Ohio, USA; 4 Department of Family Medicine, Case Western Reserve University, Cleveland, Ohio, USA

**Keywords:** diabetes, primary care, qualitative research

## Abstract

**Aim:**

To better understand the type and range of health issues initiated by patients and providers in ‘high-quality’ primary-care for adults with diabetes and low socio-economic status (SES).

**Background:**

Although quality of care guidelines are straightforward, diabetes visits in primary care are often more complex than adhering to guidelines, especially in adults with low SES who experience many financial and environmental barriers to good care.

**Methods:**

We conducted a qualitative study using direct observation of primary-care diabetes visits at an exemplar safety net practice in 2009–2010.

**Findings:**

In a mainly African American (93%) low-income population with fair cardiovascular control (mean A1c 7.5%, BP 134/81 mmHg, and low-density lipoprotein cholesterol 100 mg/dL), visits addressed a variety of bio-psychosocial health issues [median: 25 problems/visit (range 13–32)]. Physicians most frequently initiated discussions about chronic diseases, prevention, and health behavior. Patients most frequently initiated discussions about social environment and acute symptoms followed by prevention and health behavior.

**Conclusions:**

Primary-care visits by diabetes patients with low SES address a surprising number and diversity of problems. Emerging new models of primary-care delivery and quality measurement should allow adequate time and resources to address the range of tasks necessary for integrating biomedical and psychosocial concerns to improve the health of socio-economically disadvantaged patients.

## Introduction

Type 2 diabetes occurs in 1 out of 10 Americans, contributes strongly to excess morbidity and mortality, and accounts for about one in five healthcare dollars spent in the United States (CDC, 2011). African Americans and Hispanic adults have a 66–77% higher prevalence of diagnosed diabetes compared with Caucasians (CDC, 2011), and worse clinical outcomes leading to greater morbidity and mortality from diabetes (AHRQ, 2010). Adults with lower income and less education receive less recommended services (hemoglobin A1c test, dilated eye examination, and foot examination) by an absolute difference ranging from 7 to 18% between the highest and lowest status groups with subsequent worse health outcomes (Samuels *et al.*, 2008; AHRQ, 2010). Primary-care physicians are challenged to deliver high-quality preventive and disease-specific care during brief visits by patients, who often have high levels of multimorbid health conditions (Fortin *et al.*, 2005) and other diverse healthcare needs (Stange, 2009b). This struggle of delivering high-quality care efficiently is further complicated when seeing a high number of uninsured and low-income patients who experience many financial and environmental barriers to good care (Grant *et al.*, 2011).

Previous research has shown patient, provider, or system factors associated with improved diabetes outcomes, such as better medication adherence, greater medication intensification, fewer competing demands at the office visit, greater access to care, greater patient motivation, and better continuity of care (Grant *et al.*, 2007; Parchman *et al.*, 2007; Simmons *et al.*, 2007; Bolen *et al.*, 2008; 2009; Samuels *et al.*, 2008). These studies were limited in their ability to assess the context of care in individual office visits (Grant *et al.*, 2007; Bolen *et al.*, 2008; 2009; Samuels *et al.*, 2008), undercounted health issues made solely on billing diagnoses (Beasley *et al.*, 2004), had recall bias (Simmons *et al.*, 2007), and were only able to determine perceived psychosocial issues that may impact health (Simmons *et al.*, 2007). Direct observations of office visits are particularly important when assessing the type and range of health issues discussed at primary-care visits, as recall bias and medical diagnosis coding often substantially undercount the problems addressed at a visit (Beasley *et al.*, 2004). In addition, direct observation allows documentation of the psychosocial aspects of health discussed at a visit, which are often not billable or diagnosed conditions. Only one previous study has conducted in-depth observations regarding the range of health issues discussed during primary-care visits for adults with diabetes (Parchman *et al.*, 2006), but it was conducted over a decade ago, did not evaluate who initiated the health concern, and was not focused on the underserved. No study, to our knowledge, has recently described the complete range of health issues in populations with diabetes and low socio-economic status (SES). In addition, no one has evaluated the differences or similarities of health issues brought up by the patient versus the physician.

To better understand the type and range of health issues arising in ‘high-quality’ primary-care encounters with socio-economically disadvantaged adults diagnosed with diabetes, we conducted an in-depth analysis of office visits of adults with diabetes in an exemplar primary-care practice caring mainly for an African American low-income population. Given the continued and rapid changes being made in primary care, such as patient-centered medical homes and accountable care organizations, the results of this recent in-depth analysis can be used to inform policy, organization, and quality improvement efforts for clinics with socio-economically disadvantaged populations.

## Methods

### Study population

We purposefully selected one exemplar safety net clinic (defined as >50% of patients on medicaid or uninsured) from a county hospital in Northeast Ohio, performing in the top quartile on their biomedical quality of care scores for their patients with diabetes in 2009–2010 after obtaining institutional review board approval. The biomedical quality of care scores are locally adapted National Council of Quality Assurance measures for adults with diabetes, and included the percent of diabetes patients with blood pressure (BP) <140/90 mmHg, hemoglobin A1c<7%, and low-density lipoprotein cholesterol (LDL-c) <100 mg/dL. We purposively selected adult patients with diabetes who were scheduled for routine follow-up visits with their primary-care provider at the practice site.

### Data collection and management

We used participant/direct observation for data collection, as we were interested in the observed number, range, and types of health issues brought up at the primary-care visit for adults with diabetes and as the primary author was a provider at the practice site. For each of the practices’ four primary-care physicians, we audio taped, observed, and transcribed three to four routine follow-up visits with diabetes patients after obtaining informed consent from patients and physicians. We excluded the primary-care provider who authored this paper due to potential biases; however, all the other providers participated in the study. Providers were told that we were observing the visit to evaluate current primary-care practice. We continued the enrollment of patients until we reached saturation of themes (when no new themes emerged from the data analysis). The research assistant and internal medicine physician developed and piloted an observation form, environmental checklist, performed key informant interviews of providers and patients, wrote daily field notes, and composed reflective memos. For this analysis, we focused on analyzing the transcribed office visits, as these were felt to be most pertinent to our objective of evaluating the range and types of health issues that arose at the visit. The research assistant (a PhD sociology candidate) audio-recorded and observed most of the patient visits, whereas a primary-care provider/health services researcher audio-recorded and observed the remaining patient visits.

### Analysis

A multidisciplinary team used a purposeful, constant comparative approach to count and categorize health issues, raised by the patient or the physician at the primary-care visit (Bradley *et al.*, 2007). Consistent with the thematic constant comparative approach, open-coding of themes occurred first, followed by iterative revision and condensing of codes (with researchers analyzing selected transcripts and then debriefing at regular meetings), and the creation of a coding dictionary (Glaser, 1965; Boeije, 2002; Bradley *et al.*, 2007). Each visit transcript was coded by both a sociology PhD candidate and an internist, independently. They met regularly and reviewed the coding scheme and text to confirm or refute the groupings throughout the analysis. Before the start of the study, we assigned two thematic auditors to better establish validity: an experienced mixed methods researcher and family physician (K.S.) and a sociologist (A.P.). They reviewed selected transcript text, codings, and groupings. Disagreements were resolved by consensus. Our coding definitions are listed in the Appendix.

## Results

### Study population

The safety net clinic serves a mainly low-income African American population (90% African American, average median annual household income of $38 500, 83% had reported graduating from high school, 73% were women, 33% were uninsured, 26% had medicaid, 19% had medicare, and 22% were commercially insured). Out of the 20 patients asked to enroll, 15 patients consented to participate. The 15 patients were mainly African American (93%) with fair cardiovascular risk factor control (mean Hemoglobin A1c 7.5%, mean BP 134/81 mmHg, and mean LDL-c 100 mg/dL). The patients had a mixture of insurance categories: private (33%), private plus medicare (20%), medicare alone (7%), medicaid (7%), and uninsured (33%). These numbers were similar to the numbers of the overall clinic population outlined above. Patients who refused to participate (*n=*5) were more likely to be men but were otherwise similar to participants in terms of age and race. The providers were all family practice physicians, Caucasian, and married. Three providers were women and one provider was a man.

### Number, range, and type of health issues

**Figure 1:**
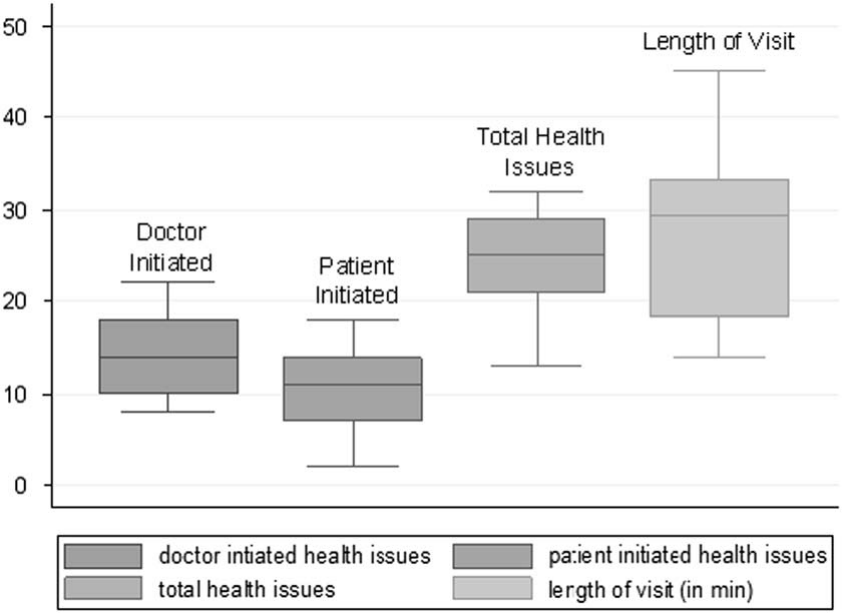
Median (interquartile range) number of health issues per visit and median visit length at 15 diabetic visits in primary care

**Figure 2:**
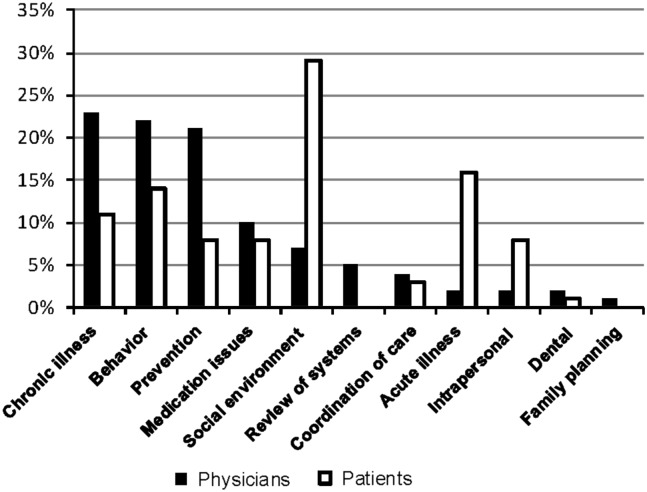
Domains of health issues initiated by clinician and patient at the primary-care visit (*n*=365 total health issues at 15 encounters)

The mean visit length was 28 min (Figure 1). A median of 25 health issues per visit were addressed (Figure 1), including a wide variety of acute and chronic medical, psychological, and social concerns (Figure 2). Primary-care physicians more frequently initiated discussions about chronic disease management and prevention (23 and 21% of 365 total health issues, respectively, at 15 patient encounters), whereas patients more frequently initiated discussions about acute health concerns and social issues related to health (16 and 29% of total health issues, respectively). The count of health issues does not include health education or personal discussions unrelated to health.

### Example case studies of routine follow-up diabetes visits

#### Description of case 1

Ms Jones has been seeing Dr Smith for many years and presented for a routine follow-up appointment. Table 1 summarizes the interaction. Dr Smith starts off discussing how Ms Jones’ recent employment has strong financial implications that will affect her health. Ms Jones confirms that she now has a job, but notes that she is not getting paid. A large part of the visit is spent discussing how to get her eight medications refilled – some with a social worker helping to fill out pharmaceutical companies’ free prescription applications and some via generic prescriptions at a local pharmacy.

**Table 1:** Case study 1: 21 healthcare issues from a patient’s 29-min primary-care visit

Coded category^a^	Illustrative direct and indirect quotations^b^	Mentioned first by
Social environment (financial)	*Dr Smith:* I heard you got a job? *Ms Jones:* Boss had no money to pay us. So happy to work but no money to pay us. [Later in the visit] *Ms Jones:* I need scripts for my social worker who is going to submit the scripts where I can get it [the meds] for free. *Dr Smith:* so you actually need a prescription for something you can take to the pharmacy since the insulin flexpens are going to be too expensive	Dr
Chronic illness (obesity)	*Dr Smith:* Hey, but you, lost 12 pounds. *Ms Jones:* I know. *Dr Smith:* That’s so good	Dr
Behavior (diet)	Ms Jones: I’m eating healthier. Ate chicken and salad. [Long discussion of her diet]	Pt
Behavior (exercise)	*Ms Jones:* I walk every day, even in the rain. *Dr Smith:* How far are you walking? *Ms Jones:* yesterday I walked from my house to chagrin boulevard where I saw you that time I was sitting at the bus stop. I also walk to that plaza	Pt
Review of systems	*Dr Smith:* Shortness of breath or chest pain when walking?	Dr
Behavior (self-monitoring)	*Ms Jones:* I invested in a (blood pressure) machine, couldn’t figure out how to use it. *Dr Smith:* We can schedule a nurse visit to do that	Pt
Behavior (self-monitoring)	*Dr Smith:* What about your sugars? *Ms Jones:* My sugars are good. I did not bring my little book but at 8 this morning it was 129 …	Dr
Social environment/larger culture (diet and exercise)	*Ms Jones:* I’ve been watching that Biggest Loser … but I can do it because the people that go on the show, they get sent home, and they do it. *Ms Jones:* And when I go to Subway, I ate subway for a whole month. Well I ate the same thing that Jared ate. *Dr Smith:* But Jared is a six foot tall guy who walked to subway twice a day.<laughter>	Pt
Behavior (medication adherence) and chronic illness (hyperlipidemia management)	*Dr Smith:* Did you start your cholesterol medication? *Ms Jones:* No. I lost the prescription. *Dr Smith:* You know that’s subconscious. You did not want that prescription. So do you want to be checked again? *Ms Jones:* yes, and I am hoping you don’t have to fuss at me. [Later] *Dr Smith*: your LDL, this was where the problem was. Your LDL was 151 and goal is <100 but we really want you <70	Dr
Chronic illness – diabetes (hypo and hyperglycemia, management, and supplies)	*Dr Smith:* Tell me what your highest sugar is. *Ms Jones:* 179. Dr Smith: and the lowest? *Ms Jones:* 82. I feel funny when my sugars are down in the 80s–90s. *Dr Smith:* your body is used to higher sugar levels, so you’re going to have to get used to what normal feels like … You don’t want to overeat to get your sugars up. [Later]*Ms Jones:* I bought those little tablet things. *Dr Smith:* Glucose tablets. *Ms Jones:* But you know what I want to invest in? One of those bands. Dr *Smith:* Medical alert bracelets. Go to medalert.com to get them. [Later] *Dr Smith:* The first part of your diabetes report is about your diabetes. So 7 or lower (for HbA1c) is where we want you. [Dr Smith reviews diabetes report card showing goals for intermediate outcomes]	Dr
Chronic illness – hypertension management	*Dr Smith*: These are your blood pressure medicines. Did you ever take lisinopril? *Ms Jones:* What is that? *Dr Smith:* a blood pressure medicine that protects your kidneys. Last time we checked your kidneys, it was fine, but that’s something we are going to have to watch	Dr
Prevention (screening)	*Dr Smith:* you had your colonoscopy but you need an eye exam. *Ms Jones:* I know I need to go. *Dr Smith:* Your PAP is due in October	Dr
Care co-ordination	*Dr Smith:* You’re due for your PAP in August and your flu shot in October. But two months late (for your PAP) is not too late. I am trying to save you a trip	Dr
Behavior (non-adherence)	*Dr Smith:* And I put the referral in last time for the eye exam so all you’ve got to do is make the appointment. *Ms Jones:* I made the appointment and then I didn’t go	Dr
Acute issue	*Ms Jones:* I need you to look at this (skin lesions on legs). *Dr Smith:* It looks more like you’ve been messing with it. *Ms Jones:* yes, I be sticking needles in them. *Dr Smith:* you need to leave it alone. Diabetics are hard to heal so don’t stick needles below your knees	Pt
Acute issue	*Dr Smith:* so what are your other concerns? *Ms Jones:* Just this, just here (Skin lesion on forehead). It hurts. [After examining lesion] *Dr Smith:* We need to send you to dermatology and let them take it off	Pt
Medication issues (refills)	*Dr Smith:* Do you need prescriptions? *Ms Jones:* I am going to need my water pill and synthroid. And insulin. [Long discussion of insulin] *Dr Smith*: Need syringes? Need lancets?	Dr
Medication issues (side effects)	*Dr Smith:* Should I write for your cholesterol medicine again? *Ms Jones:* But I got so many bottles of that Lipitor. *Dr Smith:* why can’t you take those? *Ms Jones:* Remember, it was making me sick and my joints hurt real bad	Dr
Prevention (immunizations)	*Dr Smith:* You had your tetanus, but your flu shot is due in October	Dr
Intra-personal (sexuality)	*Ms Jones:* But what if I want to schedule before four months to get a complete inside physical. I just want to get myself checked out. Cause I’m not dating no more. I have no man. I’m back to my old self	Pt
Social environment (spiritual)	*Ms Jones:* And pray for me that I get paid	Pt

Pt=patient; LDL=low-density lipoprotein.

a Categories are generally listed in the order they were discussed. Parentheses in first column are used to describe sub-categories.

b Quotations may be slightly out of order if more than one set of quotations was used to describe one coded category. We have denoted this by writing [later] for the next set of quotations.

In addition to discussions related to financial concerns, Dr Smith and Ms Jones discuss in detail her diet, exercise program, blood sugar and BP values, self-monitoring, cholesterol medication adherence, diabetic supplies (med-alert bracelets, insulin syringes, and lancets), prevention (including plans for getting her a PAP smear, colonoscopy, and an eye exam), changes in sexual partners, her spiritual beliefs as they relate to her health, and two acute issues.

#### Analysis of case 1

In-depth study reveals a rich subtext of the attitudes, beliefs, knowledge, and behaviors, as well as the role of popular culture underpinning patients’ attempts to manage their diabetes. It is instructive for ‘Dr Smith’ to know that ‘Ms Jones’ ‘ate at Subway for a whole month’ (because Jared lost weight doing so) and decided that because the contestants on the Biggest Losers maintained their weight loss ‘at home, after the show,’ she could do so as well. Ms Jones acknowledges that she allows herself ‘wiggle room’ in trying to manage her diabetes: ‘I ate only half the foot long for lunch and the other half for dinner.’ Ms Jones’ expression of the belief that ‘my body feels better when my sugar is higher’ initiated a lengthy discussion about how to manage low as well as high blood sugar.

Her physician resourcefully integrates both the physician-directed health priorities such as chronic disease management of her diabetes and the patient-directed priorities such as her social environment into the overall plan of care. For instance, the physician incorporates the patient’s concerns about potential job loss and concern for medication costs when writing prescriptions, and the provider and patient work together with the social worker to derive the most appropriate regimen that will minimize costs while maximizing adherence. In the end, the provider prescribes some expensive medications such as insulin, which will be obtained through the help of the social worker via a free pharmaceutical company program that will mail the patient her medications. Other generic medications were prescribed that cost four dollars a month, such as her cholesterol medicine. Furthermore, the physician incorporates the patient-directed comments about her social environment regarding diet into the plan for weight loss by encouraging recent weight loss while modifying the ‘subway’ diet to have less bread.

#### Other example cases

Patients and providers discussed a wide range of issues at these primary-care visits (Table 2). Importantly, the visits included a broad range of health prevention, intra-/inter-personal, and social issues that go beyond the management of chronic disease. These findings illustrate the value of addressing both biomedical and psychosocial concerns at the diabetes medical encounter and undergird the argument that delivering high-quality care requires time, subtlety, and resources.

**Table 2:** Other case examples of health-related themes brought up at several routine office visits

Health issue	Patient 1-Provider 1	Patient 2-Provider 2	Patient 3-Provider 3
Chronic illness	**Diabetes management**	**Hypertension management**	**Diabetes management**
	Provider: So what’s happening with your sugars? Patient: My sugar? Is it bad? Provider: No, I’m asking. Are you checking it regularly? Patient: every morning. Provider: Okay, and what kind of numbers are you getting? Patient: 75, 93, 102. It has not been over that	Patient: Yeah. I started taking the vinegar again and like the blood pressures been going between like, what? The other day it was like 131 over 76. Then, the next morning I was up and about and it was like 130-something over 96	Provider: How about your sugar? How is that doing, because you’re not on any medication for that, are you? Patient: No. In fact that’s the first thing they checked. It was 119 when they checked it that morning
Prevention	**Cancer screening**	**Immunizations**	**Cancer screening**
	Provider: Is your colonoscopy every five years or ten years? Patient: I think you said ten. Provider: That’s what I think too. I’m just going to double check	Provider: Yeah. And in terms of shots, you should get the flu shot this year. We don’t have it yet anyway, so I can’t even <laughter>, and I can see you shaking your head	Provider: I’m going to go ahead and give you the [stool] cards for now … And then the other screening test is the prostate. Now that’s really controversial right now, ‘cause we usually do a PSA test every year. Patient: I know
Behavior	**Checking feet**	**Medication adherence**	**Diet**
	Provider: These (feet) are beautiful. You look at them every night? Patient: My feet? Provider: Um hmm. Patient: Sometimes. <laughter> Provider: Get in the habit	Patient: So I stopped taking that one. A week after I started taking it, I almost had an accident. I was coming home, I got a cramp in my neck that went into my head, down my shoulder and stopped right at the elbow … I thought I had a stroke. Provider: Okay. So if you didn’t like the Norvasc and you didn’t like the Hydrochlorothiazide and you’re feeling like the Toprol is causing you problems, we need to find something else	Provider: So you’re just controlling that with your diet? Patient: Yes. Yes. Provider: Are you still trying to follow a, like a diabetic, I mean a really low-fat no meat diet like your wife? Patient: Yes. Yeah
Medication issues	*Provider:* The second thing I’m wondering about is that Wellbutrin you’ve been on for four years now. Provider: And what happened with the Zoloft before (‘cause we switched you)? *Patient:* I was on that for years and years and I don’t know why you took me off and gave me Wellbutrin. *Provider:* Oh we switched you ‘cause it was non-formulary	*Provider:* ‘Cause I could give you the non-generic Toprol, but it probably will be more expensive for you. *Patient:* Okay, I could try it	*Provider:* Now the thing’s rubbed off, I can’t read it, but it looks like it says you should be taking it three times a day and you’re only taking it twice a day. Do you think they want you to take it three times? *Patient:* When I first went he told me ‘You’ve got to take it three times a day,’ but after, I don’t know if my pressure went down. He only told me to take it twice I believe
Social environment	**Social support**	**Economic/financial**	**Social support**
	*Provider:* you look in that *Shaker Magazine*, there’s a whole section for seniors as far as activities. Patient: *Patient: Shaker Magazine*? I didn’t know because I went to the library in Cleveland Heights, and I asked them. I said you know ‘I live in Shaker. Do they have any activities for senior citizens?’ … But hearing it from you, I will do that	*Patient:* About two years or so, that you put me on that after I had the reaction from the Norvasc. No problems. They stopped making the generic and they said that the insurance was covering the name brand. I was taking the name brand. Remember I told you I started having a problem? Then they started making the generic again …	*Provider:* Do you have to go over there every ten days to see a nurse? *Patient:* No, I … *Provider:* So you do it yourself? *Patient:* My daughter shoots it [the medication]
Care co-ordination	*Provider:* And Dr R just did labs on you and everything that he did was okay *Patient:* I have to go and pick up the little black one [pill] from Dr R	*Provider:* Okay, and so did you get my letter with your test results from August?	*Provider:* And your heart doctor, he probably doesn’t know what happened to you, or did you tell him? *Patient:* No, I haven’t. *Provider:* Cause you might want to give them a call …
Acute illness	*Patient:* I feel tired. No energy, even though I exercise. [Long Discussion] *Provider:* So, do you think you’re depressed? *Patient:* Yep	NA	*Patient:* I just got out of the hospital a couple of … *Provider:* Oh no, ‘cause I’ve been getting letters from the heart doctor. *Patient:* Yeah. I had a slight stroke I think …
Intra-personal	**Emotional**	**Sexuality/intimacy**	NA
	*Patient:* I was very distraught when [my grandson] moved. My youngest son visits and I have two grandchildren here, you know that they visit	*Provider:* Have you been with anybody new lately? *Patient:* No	

NA=not applicable.

Sub-themes are highlighted in bold and included where a sub-theme is helpful to orient the reader.

## Discussion

Although we expected the primary-care visit for adults with diabetes and low SES to be diverse in the range of health issues, the surprisingly large number of health issues (median 25) and the inter-relationship with the social environment of patients’ lives was remarkable. Similar to our study, one previous observational study reported that primary-care physicians deal with an average of 17 topics, questions, and symptoms at the primary-care diabetic visit (Parchman *et al.*, 2006), in contrast with the average of five problems noted in the visit medical record and four problems in the bill (Beasley *et al.*, 2004).

Quantitative studies often fail to capture other issues such as the social environment (Samuels *et al.*, 2008; Martin and Sturmberg, 2009) as they relate to health, which emerged as a major domain of patient concern in our evaluation (i.e., how to afford medications, how work and family impact their diabetes management). The doctor–patient visits in this in-depth analysis highlight the large number of health and social issues addressed at the so-called routine follow-up visits between adults with diabetes and their primary-care physician in a high-quality practice of socio-economically disadvantaged patients.

These findings show what is possible in dedicated primary care – possibilities that provide hope for overcoming the chasms of fragmentation and poor quality that characterize much of the US healthcare system (IOM, 2012). Study findings provide insights that can help re-think what we mean by ‘quality’ (Krein *et al.*, 2002), which, in these visits, involve a bio-psychosocial model of care that integrates the biomedical, psychosocial, and environmental determinants of health (Engel, 1977). Excessive focus on caring for individual diseases over integrating care for the complex interactions among patients’ acute concerns, multiple chronic conditions, and psychosocial/system/environmental factors (Stange, 2009a; 2009b; 2009c) may be a reason for the poor performance of the US healthcare system compared with other countries with systems based more heavily on primary care (Schoen *et al.*, 2009). This study shows that exemplar primary-care physicians are able to integrate complex care in ways that go well beyond biochemical targets such as A1c. Although these physicians were doing well achieving their biomedical quality targets, they were also building and sustaining meaningful and caring relationships with patients, which can strongly impact patient outcomes over time (Beach and Inui, 2006; Epstein *et al.*, 2010). This caring was demonstrated by integrating care of patients’ most salient concerns, as well as their social and work lives, with collaborative management of their multiple health conditions and concerns.

Several limitations to our study deserve mention. As this was a qualitative study that was carried out at one clinic, the conclusions may not be generalizable to the overall population. However, we were not trying to generalize findings but explore the range of health issues discussed in a primary-care visit to inform future potential interventions to improve care. Second, although we tried to combat bias in our analysis by including sociologists and primary-care physicians along with third-party physician review to confirm or refute findings, we recognize that some biases are hard to fully address. For instance, providers are more likely to conclude that they need more time with patients as we have suggested in this study. However, we also note that patients are more likely to bring up psychosocial issues than providers during an office visit; therefore, not all of our findings are biased toward positive provider performance.

This study also has several important implications. First, the study raises questions about how ‘productivity’ is assessed in primary care. From bio-psychosocial and patient-centered points of view, the observed visits were highly productive. However, most of the present productivity measures and financial incentives emphasize ‘throughput’ (Morrison and Smith, 2000; Wilson *et al.*, 2006; Berenson and Rich, 2010) and place financial disincentives against caring for complex patients (Hong *et al.*, 2010). In a recent effort to increase revenue, the study site has now decreased scheduled visit time for such patients by overbooking 20 min slots based on no-show rates with no rules related to patient complexity. The average visit length of these exemplar physicians was closer to 30 min, and shows what might be possible if the complexity of visits to primary care is recognized and supported. Fortunately, some of the recent efforts to reorganize primary care (Dorr *et al.*, 2006), including some patient-centered medical home initiatives (Reid *et al.*, 2010), recognize this potential, and support smaller panel sizes, longer visits, and team support for diverse patient needs (Margolius and Bodenheimer, 2010). Second, the fact that patients were more likely to bring up psychosocial concerns compared with providers underscores the need to address these concerns in a systematic manner. Developing primary-care teams with personnel to address psychosocial concerns such as care co-ordinators and social workers is one way to better address this issue, especially for disadvantaged populations. Another mechanism might be the use of a checklist for provider teams to ensure these concerns are proactively addressed by our primary-care teams.

Systems that incentivize quality and care integration over quantity may have the benefit of allowing scheduling of complex patients at more appropriate intervals and allow for more adequate resources for caring for biomedical and psychosocial aspects of patient health. In this time of tumultuous change in care organization and payment, we should support models of primary-care delivery and quality measurement, which allow adequate time and resources to address the range and complexity of tasks that are necessary for improving the health of socio-economically disadvantaged patients.

## Appendix: coding definitions – 14 January 2011

### Chronic diseases

‘Diseases that have one or more of the following characteristics: they are permanent, leave residual disability, are caused by non-reversible pathological alteration, require special training of the patient for rehabilitation, or may be expected to require a long period of supervision, observation, or care.’^1^ A summation of the chronic diseases the patient has: diabetes, hyperlipidemia, hypertension, and other. Transient ischemic attacks (TIAs), obesity, and allergies are considered chronic conditions. If a condition is implied but not specifically mentioned/discussed, do not count it as a chronic condition. If a condition is discussed but it is a new symptom (i.e., high blood pressure (HBP) or cholesterol in a diabetic patient without a previous history of HBP or cholesterol), mark the discussion under acute/new symptom. High calcium (parathyroid hormone) and vitamin D deficits may be considered chronic conditions.

### Acute/new patient symptoms

‘Disease having a short and relatively severe course.’^2^ Any new or acute symptom, complaint, or health issue mentioned during the encounter not coded elsewhere. New chronic conditions such as TIA or stroke that has happened since the last visit and not known to the physician are considered new and may be related to diabetes (such as TIA). If a cluster of symptoms seems to be related (cough, shortness of breath, fever), then count cluster of symptoms as one new complaint NOT several. If the patient brings up a symptom very briefly (i.e., only a few words or sentence) in the midst of other complaints but it never gets discussed, then do not count it. List the symptoms discussed.

Medication issues=Medication side-effects plus medication refills described next below.

### Medication side-effects, group

This is the sum of the check blocks ‘medication side-effects, diabetes,’ ‘medication side-effects, HBP,’ ‘medication side-effects, low-density lipoprotein (LDL),’ and ‘medication side-effects, other.’ Count each instance. Include only if patient actually has the side-effects. If physician is educating the patient about possible side-effects, then code under education. If the patient is concerned about possible future side-effects and physician educates about that issue, code under education.

1. *Medication side-effects, diabetes*
  Must refer to specific side-effects of a specific diabetes medication as it affects a patient. Check only if the patient actually has side-effects.
2. *Medication side-effects, HBP*
  Must refer to specific side-effects of a specific blood pressure (BP) medication as it affects a patient; if a general comment, code under ‘education.’ Check only if the patient actually has side-effects.
3. *Medication side-effects, LDL*
  Must refer to specific side-effects of a specific LDL medication as it affects a patient; if a general comment, code under ‘education.’ Check only if the patient actually has side-effects.
4. *Medication side effects, other*
  Must refer to specific side effects of a specific medication as it affects a patient; if a general comment code under ‘education.’ Check only if the person actually has actual side-effects.

### Medication refills, overall group

If medication refills are mentioned only globally and no specific drugs are mentioned, mark the overall category of medication refills and do not mark specifics below overall category. If specific drugs are mentioned, mark who brought up medication refills first under overall category and then fill in about specific drugs below (see next few groupings on medication refills).

1. *Medication refill, diabetes*
  Specific medication and ‘refill’ must be mentioned.
2. *Medication refill, BP*
  Specific medication and ‘refill’ must be mentioned.
3. *Medication refill, LDL*
  Specific medication and ‘refill’ must be mentioned.
4. *Medication refill, other meds*
  Specific medications (other than diabetes, BP, and LDL) and ‘refill’ must be mentioned.

### (Health) behavior

‘Behaviors expressed by individuals to protect, maintain, or promote their health status. For example, proper diet and appropriate exercise are activities perceived to influence health status. Life style is closely associated with health behavior and factors influencing life style are socio-economic, educational, and cultural.’^2^ The following behaviors (defined below) are considered behaviors related to diabetes self-management: diet, exercise, monitoring feet, adherence to medications (diabetes, BP, and cholesterol), self-monitoring of sugar, self-monitoring of BP, smoking use, alcohol use, and drug use. Record behaviors relating to other health issues under ‘other behavior.’ Specify instances of patient non-adherence in the space provided.

1. *Behavior diet*
  Any reference to food, diet, or eating behavior.
2. *Behavior exercise*
  Any reference to exercise or movement of any kind, in any context; for example, a reference to ‘climbing stairs in a friend’s house’ meets the criterion.
3. *Behavior check feet*
  Specific reference about adherence, ‘Do you check your feet?’ or ‘I check my feet.’
4. *Behavior adherence, diabetes medications*
  Any reference to diabetes medications being taken. A physician merely asking a patient ‘are you taking [medication]’ meets the criterion as does a patient who mentions in passing taking a diabetes medication.
5. *Behavior adherence, BP medications*
  Any reference to BP medications being taken. A physician merely asking a patient ‘are you taking [medication]’ meets the criterion as does a patient who mentions in passing taking a BP medication.
6. *Behavior adherence, LDL medications*
  Any reference to LDL medications being taken. A physician merely asking a patient ‘are you taking [medication]’ meets the criterion as does a patient who mentions in passing taking an LDL medication.
7. *Behavior, self-monitor sugar*
  Must be specific reference to how often readings are taken, when taken, the times of day; do not infer from ancillary comments that patient is monitoring sugar. The question ‘what are your sugar levels?’ does not meet the criteria here. A discussion about needing to check sugar levels would count.
8. *Behavior, self-monitor BP*
  Can refer to frequency, timing, or be a general statement or question – for example, ‘I am monitoring my blood pressure,’ or ‘do you monitor your blood pressure at home?’ Applies if patient/provider refers to the use of a BP monitor at home.
9. *Behavior, smoking*
  Any reference to smoking. A comment or question such as ‘You are not a smoker, right?’ meets this criterion. Comments/discussions relating to cessation specifically are recorded in the ‘smoking cessation’ checkbox AND under behavior smoking here.
10. *Behavior, alcohol*
  Any reference to alcohol. A comment or question such as ‘You are not a drinker, right?’ meets this criterion. Comments/discussions relating to cessation specifically are recorded in the ‘alcohol cessation’ checkbox AND under behavior alcohol here.
11. *Behavior, drugs*
  Any reference to illegal street drugs or others. A comment or question such as ‘You are not using drugs, right?’ meets this criterion. Comments/discussions relating to cessation specifically are recorded in the ‘drug use cessation’ checkbox AND under behavior here.
12. *Behavior, other*
  Any other discussion that is felt to be related to behavior that does not fit the above categories.
13. *Non-adherence*
  Any comment that relates to patients not following the physician’s advice regarding diabetes standards and/or other healthcare issues. Include as non-adherence if a patient did not follow-up with a referral even if the referral was supposed to contact the patient. If a medication is ‘as needed’ and the patient is not taking it, this does not count as non-adherence. If the patient is not 100% adherent, then he/she would meet the non-adherence criteria, but this has to be specified in writing as a comment.
  Include as non-adherence if the patient is mentioned as not regularly seeing the physician.

### Co-ordination of care

‘We define care co-ordination as the deliberate organization of patient care activities between two or more participants (including the patient) involved in a patient’s care to facilitate the appropriate delivery of healthcare services. Organizing care involves the marshalling of personnel and other resources needed to carry out all required patient-care activities, and is often managed by the exchange of information among participants responsible for different aspects of care.’^3^ (Include references to social worker, also includes references to a physician communicating with a patient via letters.)

### Prevention

Includes sub-categories related to disease prevention like screening, immunizations, chemo preventive, and other. Preventive measures are checked even if there is only mention or discussion of them and are not actually carried out.

1. *Screening*
  Includes mention of the following: breast exam or mammogram; PAP or human papillomavirus; PSA or prostate exam; colonoscopy or fecal occult or barium; human immunodeficiency virus or other sexually transmitted diseases testing in asymptomatic individuals.
a. *Screening, other:* any mention of screening test other than those included in the definition of ‘Screening’ above. Include in this category references to lab results (e.g., blood count, liver, Hep C, etc.).
2. *Immunizations*
  Check specific immunization: tetanus, pneumonia, and flu.
a. *Immunizations, other:* immunizations other than tetanus, pneumonia, and flu.
3. *Chemo preventive*
  Includes mentions of aspirin and prenatal vitamins as well as calcium/vitamin D and ‘other’ preventive agents. Other vitamins are not included in this category.
4. *Preventive, other*
  Any preventive measure/test not included in the categories screening, immunizations, and chemo preventive.

### Review of systems

Any physical exam/mention of/discussion of system – for example, respiratory, nervous, reproductive, circulatory, digestive, etc. The question ‘Have you had any chest pain and/or shortness of breath’ meets this criterion.

### Psychosocial variables, group (intra-personal and social environment)

The coding scheme for this project reflects the World Health Organization’s definition of ‘health’: ‘Health is a state of complete physical, mental, and social well-being and not merely the absence of disease or infirmity.’^4^ Specification of the following nine categories was data-driven; that is, these themes, falling under the psychosocial rubric, emerged from the data. We further classified the nine variables into two groups: Intra-personal (sexuality, intimacy; emotional) and social environment (community, neighborhood; larger culture, society; economic, financial, insurance; work, occupation, job; family social connections; non-family social connections; spiritual, religious.) ‘Intra-personal’ is defined as ‘occurring inside a person’s mind or character;’^5^ and ‘social environment’ is defined as variables encompassing ‘the immediate physical surroundings, social relationships, and cultural milieu within which defined groups of people function and interact.’^6^ Attempt to record for each category only those references that relate to health issues; however, if in doubt, assume the issue is health-related.

### Social environment – neighborhood, community

Include any references to community, local resources such as other health system resources (e.g., availability of discounted medications at a system clinic). Does not include mentions/discussion of Partners in Care and Medicare; these are included in the financial category, also includes references to healthcare related issues such as neighborhood crime, availability of healthcare services, Urgent Care, other medical centers, etc.

### Social environment – larger culture, society

By ‘society’ we mean a geographical territory – the United States – that is distinguished by its cultural, structural, and population/ecological characteristics.^7^ Include references to health-related cultural or societal phenomena – for example, eating at Subway to lose weight; watching Biggest Losers and adopting its spreadsheet system to track weight loss; ubiquity of fast food restaurants; and public policy as it relates to healthcare. Place references to insurance status or other financial concerns in the financial category. Do not include here (put in economic category) references to larger cultural economic issues – that is, difficult labor market, etc.

### Social environment – economic, financial, and insurance

Include references to the patient’s financial situation as it relates to health matters – for example, ability to afford medical care, prescriptions, insurance status, etc. Also include here references to the larger societal issue of unemployment, difficulty finding jobs.

### Social environment – work, occupation, and job

Include health-related references to work, jobs, occupation, and retirement.

### Social environment – family social connections

Include health-related references to family – for example, daughter checks my BP, caregiver for ill relative, etc.

### Social environment – non-family social connections

Health-related social connections not included in the family member connection category – for example, difficult to follow diet when out with friends.

### Social environment – spiritual, religious

Include references to a religion, church, deity, spirituality in relation to health. Record statements such as ‘pray for me.’

### Intra-personal – sexuality, intimacy

Include health-related issues involving intimate partner – for example, sexual activity discussed in relation to discussion of birth control.

### Intra-personal – emotional

Include reference to mental ‘feeling’ or ‘affection’ (e.g., of pleasure or pain, desire or aversion, surprise, hope or fear, etc.), as distinguished from cognitive or volitional states of consciousness.’^8^ Record any expression of emotions related to health for example, missed appointments because grieving deaths. Does NOT include discussion of depression or anxiety symptoms. If there is a history of depression and discussing this, then list under chronic conditions. If no history of depression, but discussing new symptoms of depression or anxiety that needs medical attention, then classify under acute/new symptoms.

### Dental

Any comment/reference made to dental health.

### Family planning

Any discussion related to birth control or pregnancy.

### References for coding definitions

1. PubMed, MeSH, *Dictionary of health services management*, second edition. Retrieved from http://www.ncbi.nlm.nih.gov/mesh

2. PubMed, MeSH, http://www.ncbi.nlm.nih.gov/mesh

3. McDonald, K.M., Sundaram, V., Bravata, D.M., Lewis, R., Lin, N., Kraft, S., McKinnon, M., Paguntalan, H., Owens, D.K. 2007: Care coordination. In Shojania, K.G., McDonald, K.M., Wachter, R.M., Owens, D.K., editor, *Closing the quality gap: a critical analysis of quality improvement strategies. Technical review 9* (Prepared by the Stanford University-UCSF Evidence-based Practice Center under contract 290-02-0017: AHRQ Publication No. 04(07)-0051-7), Volume 7. Rockville, MD: Agency for Healthcare Research and Quality.

4. Preamble to the Constitution of the World Health Organization as adopted by the International Health Conference, New York, 19–22 June, 1946; signed on 22 July 1946 by the representatives of 61 States (Official Records of the World Health Organization, no. 2, 100pp.) and entered into force on 7 April 1948.

5. Intrapersonal 1989: *The Oxford english dictionary*, second edition. OED Online. Oxford University Press. Retrieved 5 January 2011 from http://www.oed.com/view/Entry/98580?redirectedFrom=intrapersonal#eid143972

6. Barnett, E., Casper, M. 2001: A definition of ‘social environment’ (letters). *American Journal of Public Health* 91, 465.

7. Johnson, A.G. 1995: *Blackwell dictionary of sociology*. Massachusetts Blackwell Publishers, Inc., 268 pp.

8. Emotion 1989: *The Oxford english dictionary*, second edition. OED Online. Oxford University Press. Retrieved 5 January 2011 from http://www.oed.com/view/Entry/61249?redirectedFrom=emotion
